# Continuity of care for newly diagnosed diabetic patients: A population-based study

**DOI:** 10.1371/journal.pone.0221327

**Published:** 2019-08-22

**Authors:** Ying-Chun Li

**Affiliations:** Institute of Health Care Management, National Sun Yat-sen University, Kaohsiung, Taiwan; Osakidetza Basque Health Service, SPAIN

## Abstract

This study explores whether continuity of care is associated with health care outcomes and medical care use among patients with newly diagnosed diabetes. A retrospective cohort analysis was performed using the Taiwanese National Health Insurance database, and cases were followed up from January 2010 to December 2012. Four thousand and seven patients with newly diagnosed diabetes were followed for 3 years. The continuity of care was measured using the continuity of care index (COCI) and the usual provider continuity score (UPCS) with high and low dichotomous categories. The probabilities of dementia, hospitalization, emergency room visits, and death were used as health care outcomes. Medical care use was defined as the number of hospital admissions, length of hospital stays, and number of emergency room visits. Adjusted odds ratios (ORs) were obtained using multivariate logistic regression; adjusted ORs for the probabilities of dementia, hospital admissions, and emergency room visits in the higher COCI patient group were 0.582 (p < 0.05), 0.623 (p < 0.001), and 0.650 (p < 0.001), respectively. Negative binomial regression models for medical resource use indicated that the group with higher COCI scores used fewer medical resources compared with the group with lower COCI scores. The findings of UPCS analysis showed that those in the high COCI group also fell into the high UPCS group. In this study, continuity of care was associated with favorable health care outcomes and less medical care uses among newly diagnosed diabetic patients. Long-term relationships between patients and health care providers should be enhanced to provide improved continuity of care.

## Introduction

The prevalence of chronic disease continues to increase [1, 2] and is exacerbated by aging populations, which burdens health care delivery systems and societies. Many older people have multiple chronic diseases [1, 3], which increase the challenges of providing efficient care to these patients. Increasingly, providers see patients at various institutions and locations, which raises concerns regarding care fragmentation [4]. In patients with chronic diseases, fragmented or discontinuous care can have a significantly negative effect on treatment outcomes [5]. Furthermore, researchers have reported more efficient medical care and enhanced health outcomes, such as low mortality and hospitalization rates, after improving continuity of care in patients with chronic diseases [5–7].

Diabetic patients consume considerable health care resources and place a substantial burden on health care delivery systems [8], and the number of diabetic patients continues to increase [9]. Diabetic patients have a high risk of dementia, worsening their health care outcomes [10, 11]. Diabetes is associated with gradually progressive end-organ damage in the brain. The brain complication is referred to as “diabetic encephalopathy” which increases the risk of dementia [12]. Continuity of care has been shown to enhance health care outcomes across several medical fields [13–16]. However, few studies have investigated whether continuity of care improves health care outcomes for newly diagnosed diabetic patients. Thus, this study applied a representative national database to explore the relationship between diabetic health care outcomes and continuity of care.

Continuity of care studies have applied different methodologic approaches and investigated various patient populations [6]. However, many studies have implemented cross-sectional approaches with a small number of patients in a specific age group [13, 17], which makes generalizations problematic. Due to the lack of a strict referral system in Taiwan, patients can visit any physician, which can lead to patients that frequently switch providers; a phenomenon called “doctor-shopping” [18], which can result in discontinuity of care. In addition, studies looking at major factors that affect continuity of care for chronic diseases have been inconclusive [19–21], and further investigations are required to improve health care delivery systems. Another study explored the benefits of continuity of care for outcomes and quality of care among diabetic patients and revealed inconsistent results [22].

In the present study, we aimed to answer the following research questions to address gaps in continuity of care research:

1. What are the associations between continuity of care and the use of medical resources for newly diagnosed diabetic patients?
2. What are the outcomes of continuity of care for newly diagnosed diabetic patients?

Answers to these questions can provide valuable insights for both research and policy-making decisions. The results of this study can provide empirical evidence for policymakers and researchers to implement continuity of care for more efficient disease management of diabetic patients.

## Methods

Data were obtained from the National Health Insurance (NHI) database, which includes 1 million observations. The NHI database is a random sample selection from the entire Taiwanese population (23 million people). Diabetes mellitus cases were first identified based on the International Classification of Diseases, 9th Revision, Clinical Modification code 250 in the baseline year of 2009. Patients younger than 45 years of age were excluded. Patients that were diagnosed with dementia before diabetes was diagnosed in 2009 were excluded. Cases with fewer than four outpatient visits related to diabetes were excluded. Diabetic cases diagnosed before 2009 were also excluded. In 2009, 4007 diabetic cases were new diagnosed. Each observation was followed-up for 3 years from January 2010 to December 2012.

Primary health care outcomes were the probabilities of dementia, hospitalizations, emergency room (ER) visits, and deaths during the study period. The primary measurements of medical care use were the number of hospital admissions, length of hospital stays, and number of ER visits.

Continuity of care levels, which were assessed using various methods [23], were key independent variables. In the present study, two separate metrics were applied to evaluate continuity of care. First, the continuity of care index (COCI) is widely used to measure the personal continuity of care in terms of the number of physician visits and the total number of physicians [24, 25]. This index score reflects a physician’s share of a patient’s total visits (the Herfindahl–Hirschman index). When patients visit fewer providers, the score is higher [24, 26]. The COCI can be expressed as follows:

$$COC\;Index=\frac{\sum_{j=1}^{M}n_{j}^{2}-N}{N(N-1)}$$

where *M, n_j_*, and *N* denote the total number of physicians, the number of visits to a physician *j*, and the total number of physician visits, respectively.

COCI scores are between 0 and 1. A score of 1 was obtained if a patient’s care visits were with the same physician, and a score of 0 is obtained if all of the visits were with different physicians. The COCI is advantageous because it reflects the distribution of care visits without being restricted to specific providers.

Second, the usual provider continuity score (UPCS) was used to evaluate the density of care patterns. In the UPCS, the health care provider with the most visits is determined, and the result is the proportion of visits with that provider [6, 27]. The UPCS can be expressed as follows:

$$\frac{n}{N}$$

where *N* and n are the total number of visits and the greatest number of visits with physicians that made the most visits, respectively. The strength of this method is in its simplicity.

When patients have a low number of visits, it can be difficult to determine the continuity of care accurately. In cases with only one, two, or three visits, the minimum or maximum continuity of care score (0 or 1) could easily be obtained. Therefore, studies have recommended restricting analyses to patients with more than three visits [3, 6]. The continuity indices were grouped into high and low groups. The high and low continuity groups were defined based on score distributions. Continuity scores in the upper 50% were defined as the high continuity group and those in the lower 50% were defined as the low continuity group. The approach of dichotomizing the variables of continuity of care was according to a previous study [28], which divided COCI into high and low groups based on median distribution values. This approach of defining binary covariates could be preferred because it offers a simpler interpretation of common effect measures from statistical models such as odds ratios [29].

The study analysis also controlled for two groups of essential covariates, namely the medical care institution and individual demographic characteristics. We included age and sex in demographic characteristics because previous studies indicated that the use of health care resources and health care outcomes could vary between different ages and sexes [30]. The Charlson comorbidity index (CCI) [31, 32] was used as a proxy measurement of individual health status. Medical care institutions were categorized as medical centers, regional hospitals, district hospitals, and clinics according to their function and accreditation level. Additionally, institution responses to competition, market opportunities, and government regulations and policies tended to vary with ownership type. Moreover, financial considerations and objectives differed between teaching and nonteaching institutions. Teaching hospitals typically provided care to indigent and high-cost patients and functioned as regional referral centers with the addition of research and education [33]. The characteristics of medical care institutions have a considerable effect on health care. Therefore, medical care institution characteristics were included in the analysis model. Factors due to which patients most frequently visited a facility were defined as medical care institution characteristics. If two or more facilities had a tie for the number of visits, the most recently visited medical care facility was used to indicate medical facility characteristics. The number of physician visits was also divided into low, intermediate, and high groups following the findings of previous studies [28, 34, 35], and each group included one-third of the total number of physician visits.

In the current study, descriptive analyses were first conducted to evaluate the characteristics of studied variables. Summary results such as the means, standard deviations, and percentages were reported. Statistical significances were determined based on chi-square and t-test analyses that determined whether patient demographics, the relationship of continuity of care with medical care use, and the relationship of continuity of care with health care outcomes differed between high and low continuity groups. Then, the relationship of continuity of care and health care use as well as health care outcomes was estimated through inferential analysis. Multivariate logistic regression was used to predict how the continuity of care affects the probabilities of health care outcomes. Negative binomial regression was also used to determine the effect of continuity of care on medical care use. Regression models were adjusted for individual characteristics and medical care institution factors.

The National Health Insurance Research Database consists of anonymous public data released for research purposes. This study conformed to the ethical standards established by the Declaration of Helsinki and was approved by the Institutional Review Board of Yuan’s General Hospital (20150323C) in Taiwan.

## Results

The final analyses included 4,007 observations. Both the high and low COCI groups contained approximately 50% of observations (Table 1). The average COCI scores were 0.982 and 0.442 in the high and low COCI groups, respectively. The samples contained more men than women. The mean age in the high COCI group (61.092 years) was slightly higher than that in the low COCI group (60.773 years). Most of the observations (>60%) were in the group of patients aged 45–64 years. Compared with the low COCI group, the high COCI group had more observations with a CCI score of 0 and fewer observations with the highest CCI score. There was also a higher proportion of older patients having a CCI score of 1 or >2. The proportion of patients visiting hospitals in urban areas was higher in the high COCI group than in the low COCI group and vice versa. Most patients visited private institutions (>46%); clinics were the most frequently visited, followed by regional hospitals, medical centers, and district hospitals. The majority of patients visited nonteaching hospitals (>51%). Medical facility characteristics referred to those which patients most frequently visited. The sample distributions were similar for the UPCS measurements.

**Table 1:** Characteristics of study samples.

Characteristics	COCI(N = 4,007)		UPCS(N = 4,007)	
	Lower COCI	Higher COCI p-value	Lower UPCS	Higher UPCS p-value
**Sample size**	2,008 (50.11%)	1999 (49.89%)	2,012 (50.21%)	1,995 (49.79%)
**Average Score**(M±SD)	0.442±0.184	0.982±0.052 <0.001	0.571±0.196	0.990±0.028 <0.001
**Sex**				
Women	924 (46.02%)	922 (45.97%) 0.946	925 (46.25%)	921 (46.17%) 0.903
Men	1,084 (53.98%)	1,077 (54.03%)	1,087 (53.75%)	1,074 (53.83%)
**Age**				
Age (M±SD)	60.773±10.469	61.092±10.550 0.337	60.781±10.466	61.085±10.554 0.361
45~64	1,334 (66.43%)	1,297 (64.88%) 0.681	1,336 (66.40%)	1,295 (64.91%) 0.688
65~74	419 (20.87%)	439 (21.96%)	420 (20.87%)	438 (21.95%)
75~84	224 (11.16%)	226 (11.31%)	225 (11.18%)	225 (11.28%)
Over 85	31 (1.54%)	37 (1.85%)	31 (1.54%)	37 (1.85%)
**CCI Score**				
0	820 (40.84%)	1,042 (52.13%) <0.001	821 (40.81%)	1,041 (52.18%) <0.001
1	494 (24.60%)	505 (25.26%)	495 (24.60%)	504 (25.26%)
Over 2	694 (34.56%)	452 (22.61%)	696 (34.59%)	450 (22.56%)
**Urbanization Level of Hospital Area**				
Level 1 (Highest)	619 (30.83%)	639 (31.97%) 0.199	620 (30.82%)	638 (31.98%) 0.190
Level 2	726 (36.16%)	753 (37.67%)	728 (36.18%)	751 (37.64%)
Level 3	255 (12.70%)	214 (10.71%)	256 (12.72%)	213 (10.68%)
Level 4 (Lowest)	408 (20.32%)	393 (19.66%)	408 (20.28%)	393 (19.70%)
**Hospital Ownership**				
Public	474 (23.61%)	457 (22.86%) 0.270	475 (26.61%)	456 (22.86%) 0.236
Private	967 (48.16%)	931 (46.57%)	910 (48.21%)	928 (46.52%)
Non-profit Proprietary	567 (28.24%)	611 (30.57%)	567 (28.18%)	611 (30.63%)
**Hospital Type**				
Medical Center	398 (19.82%)	399 (19.96%) 0.896	399 (19.83%)	398 (19.95%) 0.906
Regional Hospital	522 (26.00%)	536 (26.81%)	523 (25.99%)	535 (26.82%)
District Hospital	299 (14.89%)	284 (14.21%)	299 (14.86%)	284 (14.24%)
Clinic	789 (39.29%)	780 (39.02%)	791 (39.31%)	778 (39.00%)
**Teaching Status**				
Non-teaching	1,041 (51.84%)	1,022 (51.13%) 0.650	1,043 (51.84%)	1,020 (51.13%) 0.652
Teaching	967 (48.16%)	977 (48.87%)	969 (48.16%)	975 (48.87%)

Note: The distributions of individual factors and medical care facility characteristics between Higher/Lower COCI or Higher/Lower UPCS were not statistically significant, except for CCI score.

The high COCI and UPCS groups exhibited considerably lower medical care use, including fewer hospitalizations, shorter stays, and fewer ER visits, compared with low COCI and UPCS groups (Table 2). The high COCI and UPCS groups had fewer adverse outcomes, including fewer instances of dementia, hospital admissions, ER visits, and deaths, compared with low COCI and UPCS groups (Table 3).

**Table 2:** Relationship of continuity of care with medical care use.

Variable	COCI			UPCS		
	Lower COCI	Higher COCI	*p*-value	Lower UPCS	Higher UPCS	*p*-value
**Numbers of** **Hospitalizations** (M±SD)	1.291±2.553	0.880±2.441	<0.001***	1.306±2.618	0.864±2.367	<0.001***
**Length of** **Hospitalizations** (M±SD)	9.993±12.606	8.510±12.200	0.0185*	9.995±12.591	8.502±12.218	0.0178*
**Numbers of** **ER Visits** (M±SD)	1.838±3.627	1.163±2.431	<0.001***	1.837±3.625	1.162±2.432	<0.001***

* p < 0.05

** p < 0.01

***p<0.001

**Table 3:** Relationship of continuity of care with health care outcomes.

	COCI			UPCS		
	Lower COCI	Higher COCI	*p*-value	Lower UPCS	Higher UPCS	*p*-value
**Dementia**			0.013*			0.008**
No	1,949 (97.06%)	1,964 (98.25%)		1,952 (97.02%)	1,961 (98.30%)	
Yes	59 (2.94%)	35 (1.75%)		60 (2.98%)	34 (1.70%)	
**Admission**			<0.001***			<0.001***
No	1,084 (53.98%)	1,321 (66.08%)		1,085(53.93%)	1,320 (66.17%)	
Yes	924 (46.02%)	678 (33.92%)		927 (46.07%)	675 (33.83%)	
**ER visits**			<0.001***			<0.001***
No	830 (41.33%)	1,064 (53.23%)		832 (41.35%)	1,062 (53.23%)	
Yes	1,178 (58.67%)	935 (46.77%)		1,180 (58.65%)	933 (46.77%)	
**Death**			0.058			0.062
No	1,865 (92.88%)	1,886 (94.35%)		1,869 (92.89%)	1,882 (96.44%)	
Yes	143 (7.12%)	113 (5.65%)		143 (7.11%)	113 (5.66%)	

* p < 0.05

** p< 0.01

***p<0.001

The high COCI and UPCS groups had significantly lower probabilities of adverse health care outcomes compared with the low COCI and UPCS groups according to logistic regression models (Table 4). For the probability of dementia in the higher COCI group, the crude odds ratio (OR) and adjusted OR were 0.589 (p < 0.05) and 0.582 (p < 0.05), respectively. This model was adjusted for age, sex, CCI (Charlson comorbidity index) scores, the urbanization level of where the hospitals were located, hospital ownership, hospital type, hospital teaching status, and the number of physician visits. For the probability of hospital admissions, the crude and adjusted ORs for the high COCI group were 0.602 (p < 0.001) and 0.623 (p < 0.001), respectively. For the probability of ER visits, the crude and adjusted ORs for the high COCI group were 0.619 (p < 0.001) and 0.650 (p < 0.001), respectively. For the probability of death, the crude and adjusted ORs for the high COCI group were 0.781 and 0.863, respectively, but these results were not significant. Results of the analyses for the high UPCS and high COCI groups were similar.

**Table 4:** Crude and adjusted odds ratios organized by health care outcome during 3-year follow-up.

	COCI (N = 4,007)				UPCS (N = 4,007)			
	Lower COCI		Higher COCI		Lower UPCS		Higher UPCS	
	n = 2,031		n = 2,017		n = 2,035		n = 2,013	
**Health Care Outcome**	n	(%)	n	(%)	n	(%)	n	(%)
**Dementia**	59	(2.94)	35	(1.75)	60	(2.98)	34	(1.70)
Crude OR (95% CI)	1		0.589* (0.386, 0.899)		1		0.564** (0.369, 0.863)	
Adjusted OR (95% CI)	1		0.582* (0.375, 0.904)		1		0.561** (0.361, 0.873)	
**Admission**	924	(46.02)	678	(33.92)	927	(46.07)	675	(33.83)
Crude OR (95% CI)	1		0.602*** (0.530, 0.684)		1		0.599*** (0.527, 0.680)	
Adjusted OR (95% CI)	1		0.623*** (0.543, 0.716)		1		0.621*** (0.540, 0.713)	
**ER Visits**	1,178	(58.67)	935	(46.77)	1,180	(58.65)	933	(46.77)
Crude OR (95% CI)	1		0.619*** (0.546, 0.702)		1		0.619*** (0.547, 0.702)	
Adjusted OR (95% CI)	1		0.650*** (0.570, 0.741)		1		0.651*** (0.571, 0.743)	
**Death**	143	(7.12)	113	(5.65)	143	(7.11)	113	(5.66)
Crude OR (95% CI)	1		0.781 (0.606, 1.001)		1		0.785 (0.608, 1.013)	
Adjusted OR (95% CI)	1		0.863 (0.657, 1.132)		1		0.869 (0.662, 1.141)	

* p < 0.05

** p < 0.01

***p < 0.001

*Note*: Logistic regression models were adjusted for age, sex, the Charlson comorbidity index score, the urbanization level of where the hospitals were located, hospital ownership, hospital type, hospital teaching status, and the number of physician visits.

Negative binomial regression models of medical resource use indicated that the high COCI group had a significantly lower incidence rate ratio (IRR) for the number of hospitalizations, length of hospitalizations, and number of ER visits than did the low COCI group (p < 0.001; Table 5). For individual factors, men had a higher IRR for the number of hospitalizations (p < 0.001), length of hospitalizations (p < 0.001), and number of ER visits (p < 0.01). Older age was also associated with increased IRRs for length of hospitalization, and the number of hospitalizations and ER visits. Patients with higher CCI scores had increased IRRs for hospitalization length as well as the number of hospitalizations and ER visits. Patients who attended hospitals in the lowest urbanized areas had higher IRRs for the number of hospitalizations (p < 0.001) and number of ER visits (p < 0.001) compared with those who attended hospitals in highly urbanized areas. Patients who mostly attended private hospitals had higher IRRs for the number of hospitalizations than did those who mostly attended public hospitals (p < 0.05). Patients who mostly attended clinics had lower IRRs for the number of hospitalizations than did those who mostly attended medical centers (p < 0.001). Additionally, compared with patients who mostly attended medical centers, those who mostly attended other types of hospitals had lower IRRs for the length of hospitalizations. Patients who mostly attended teaching hospitals had higher IRRs for the number of ER visits (p < 0.01) compared with those who mostly attended nonteaching hospitals. The high group for the number of physician visits had significantly higher IRRs for the number of hospitalizations, length of hospitalizations, and number of ER visits than did the low group for the number of physician visits (p < 0.001). All analytical results and significances in the high UPCS group showed similar trends.

**Table 5:** Analysis of continuity of care and medical care use through negative binomial regression models during 3-year follow-up.

Variable	COCI			UPCS		
	Numbers of Hospitalizations (IRRs)	Length of Hospitalizations (IRRs)	Numbers of ER Visits (IRRs)	Numbers of Hospitalizations (IRRs)	Length of Hospitalizations (IRRs)	Numbers of ER Visits (IRRs)
**COCI / UPC** (Ref : Lower)						
Higher	0.75*** (0.67, 0.83)	0.61*** (0.52, 0.72)	0.68*** (0.62, 0.75)	0.74*** (0.66, 0.82)	0.60*** (0.51, 0.71)	0.69*** (0.63, 0.75)
**Sex** (Ref : Female)						
Men	1.39*** (1.24, 1.55)	1.35*** (1.14, 1.60)	1.15** (1.05, 1.26)	1.38*** (1.24,1.55)	1.34** (1.13, 1.59)	1.15** (1.05, 1.26)
**Age** (Ref : 45~65)						
65~74	1.46*** (1.28, 1.67)	1.82*** (1.48, 2.23)	1.02 (0.91, 1.15)	1.46*** (1.28, 1.67)	1.82*** (1.49, 2.24)	1.02 (0.91, 1.15)
75~84	1.92*** (1.63, 2.27)	2.72*** (2.08, 3.56)	1.47*** (1.28, 1.68)	1.92*** (1.62, 2.26)	2.72*** (2.08, 3.55)	1.46*** (1.27, 1.68)
Over 85	3.07*** (2.12, 4.44)	6.18*** (3.26, 11.72)	3.29*** (2.44, 4.45)	3.07*** (2.12,4.44)	6.15*** (3.24, 11.66)	3.29*** (2.44, 4.45)
**CCI Scores** (Ref : 0)						
1	1.28*** (1.11, 1.48)	1.55*** (1.26, 1.91)	1.31*** (1.17, 1.47)	1.28*** (1.11, 1.48)	1.55*** (1.26, 1.90)	1.32*** (1.17, 1.48)
Over2	2.13*** (1.87, 2.43)	3.04*** (2.47, 3.73)	1.65*** (1.48, 1.84)	2.13*** (1.86, 2.42)	3.00*** (2.44, 3.69)	1.65*** (1.48, 1.84)
**Urbanization Level of Hospital Area** (Ref : Level 1: Highest)						
Level 2	1.02 (0.89, 1.17)	1.15 (0.94, 1.40)	1.08 (0.96, 1.20)	1.02 (0.89, 1.17)	1.15 (0.94, 1.40)	1.08 (0.96, 1.20)
Level 3	1.48*** (1.21, 1.80)	1.96*** (1.46, 2.63)	1.31*** (1.11, 1.54)	1.47*** (1.20, 1.79)	1.93*** (1.43, 2.58)	1.31*** (1.11, 1.54)
Level 4 (Lowest)	1.43*** (1.21, 1.69)	1.59*** (1.23, 2.05)	1.30*** (1.13, 1.50)	1.43*** (1.21, 1.69)	1.59*** (1.23, 2.05)	1.30*** (1.14, 1.50)
**Hospital Ownership** (Ref : Public)						
Private	1.24* (1.05, 1.46)	1.04 (0.80, 1.35)	1.14 (0.99,1.31)	1.23* (1.05, 1.46)	1.04 (0.80, 1.35)	1.14 (0.99, 1.31)
Non-profit Proprietary	0.94 (0.81, 1.09)	0.80 (0.63, 1.00)	0.97 (0.85, 1.09)	0.94 (0.81, 1.09)	0.80 (0.63, 1.01)	0.97 (0.85, 1.09)
**Hospital Type** (Ref : Medical Center)						
Regional Hospital	1.05 (0.89, 1.23)	0.95 (0.74, 1.23)	0.99 (0.87, 1.14)	1.05 (0.89, 1.23)	0.95 (0.74, 1.23)	0.99 (0.87, 1.14)
District Hospital	0.70* (0.51, 0.95)	0.47*** (0.30, 0.74)	1.01 (0.78,1.32)	0.70* (0.51, 0.95)	0.47*** (0.30, 0.75)	1.01 (0.78, 1.32)
Clinic	0.36*** (0.25, 0.52)	0.23*** (0.13, 0.40)	0.74 (0.54, 1.01)	0.36*** (0.25, 0.52)	0.23*** (0.13, 0.40)	0.74 (0.55, 1.01)
**Teaching Status** (Ref : Non-teaching)						
Teaching **Number of Physician Visits** (Ref: Low) Intermediate High	1.11 (0.82, 1.50) 1.61*** (1.39, 1.85) 2.43*** (2.11, 2.80)	0.82 (0.52, 1.27) 1.69*** (1.37, 2.09) 2.41*** (1.96, 2.98)	1.42** (1.09, 1.85) 1.43*** (1.27, 1.60) 2.39*** (2.12, 2.68)	1.11 (0.82,1.50) 1.60*** (1.39, 1.85) 2.43*** (2.11, 2.79)	0.82 (0.52, 1.27) 1.68*** (1.36, 2.07) 2.41*** (1.95, 2.97)	1.42** (1.09, 1.85) 1.42*** (1.27, 1.60) 2.39*** (2.12, 2.68)

* p < 0.05

** p < 0.01

*** p < 0.001

## Discussion

The results of this study indicated that continuity of care is associated with health care outcomes and medical care use among newly diagnosed diabetic patients. The probabilities of dementia, hospital admissions, and ER visits were significantly lower among those with higher continuity of care. The higher continuity of care group exhibited significantly fewer hospitalizations, shorter lengths of hospital stays, and fewer ER visits compared with the group with lower continuity of care. The results were consistent with the findings of previous studies [5, 36–40]. A study of Korean patients indicated that the probability of hospital admission for patients with type 2 diabetes could be reduced through continuity of care [36]. In the United States, patients with chronic diseases, such as diabetes, were more likely to receive appropriate continuity of care [40]. Moreover, in Korea, the efficiency of health care spending and quality of care were affected by the continuity of care among diabetic patients [5]. In Taiwan, a significant inverse relationship was observed between the continuity of diabetic patient care and the risk of hospital admission for complications arising from diabetes over a long period [37]. A Canadian study showed that the higher continuity of care group had lower rates of hospitalization and death compared with the lower continuity of care group [39]. In addition, a Korean study found that continuity of care was negatively associated with all-cause and cardiovascular mortality, the number of inpatient and outpatient days, health care costs, and cardiovascular events among newly diagnosed diabetic patients [38].

With universal health care coverage and no strict referral system in Taiwan, patients are free to choose preferred providers and can switch providers frequently, which thus increases the risk of having a poor continuity of care with care fragmentation. If new diabetic patients could have good continuity of care at an early disease stage, health care outcomes and medical care uses could be improved. However, previous studies looking at continuity of care in diabetic patients have seldom focused on newly diagnosed diabetic patients. Therefore, our study has helped to fill this research gap.

In the present study, we applied two measurements for continuity of care, namely the COCI and UPCS. These measurements had similar effects on health care outcomes and medical care use, indicating that such measurements were comparable in this study. Nevertheless, previous studies have shown the COCI had marginally better power in being able to evaluate and explain patient care outcomes [36].

The prevalence of dementia is well-recognized to be higher in diabetic patients than non-diabetic patients [41]. Type 2 diabetes is associated with gradually progressive end-organ damage not only to the eyes and kidneys but also to the brain. Brain complications are characterized by mild to moderate impairments in cognitive functioning, which is referred to as “diabetic encephalopathy.” There is also an increased risk of dementia [12]. It is critical to detect moderate cognitive dysfunction early so that risk factors can be reduced, which should result in the prevention of dementia [41]. For patients with newly diagnosed diabetes, good continuity of care could provide early detection of cognitive dysfunction, which could result in physicians providing necessary treatments to prevent severe cognitive dysfunction, such as dementia. In Taiwan, an increasing prevalence of diabetes and dementia has occurred in recent years [42], which has led researchers to understand the importance of this association. It has also led to more interest in studying this association.

To assess the relationship between the frequency of adverse events and the continuity of care, larger studies with more participants are needed [43]. In the present study, a representative national data set of 1 million observations was applied, which represented a considerable increase in sample size compared with previous studies. Therefore, the results of this study are more robust.

The number of hospitalizations, length of hospital stays, and the number of ER visits increased with age. In particular, patients aged older than 85 years used the most medical care. Age is a critical factor that affects health care outcomes and medical care use. A Korean study showed that older patients with appropriate continuity of care had lower health care costs and reduced risks of hospitalization and ER visits [44]. A Canadian study that looked at older diabetic patients indicated that a higher continuity of family physician care was associated with reductions in the likelihood of hospitalization and death [39]. Moreover, patients with CCI scores greater than 2 used significantly more medical care. The presence of additional comorbidities could be associated with the need for more health care services, thus increasing the use of medical care. In this study, patients that were older than 85 years had more CCI scores of >2; therefore, these patients might have had greater medical care requirements.

The characteristics of the medical care facility did not have a strong effect on medical care use. Two potential explanations for this phenomenon are presented. First, the NHI program was implemented in Taiwan in 1995. Since the program’s inception, access to care has substantially improved, financial barriers have been minimal, and people can receive health services from various types of medical care facilities. Second, because Taiwan does not have a strict referral system, patients can choose health care providers based on their preferences. Therefore, because patients have access to the medical care facility of their choice, the characteristics of medical care facilities had limited influence on medical care use.

For the treatment of diabetic patients, spending efficiencies and qualities of care are improved by continued treatment from a single doctor [5]. Older adults who visit several different physicians tend to have poor outcomes due to an inefficient transfer of information between the various care providers as well as other care delivery deficiencies [4, 45]. This situation raises major concerns regarding the efficient coordination of care because high interpersonal continuity is related to enhanced outcomes in terms of primary and secondary clinical targets among diabetic adults [46]. Another research study encouraged policymakers to enhance the continuity of care by strengthening the patient-provider relationship to reduce hospitalizations [37]. Clinicians and policymakers involved in patient care should develop and implement programs that can improve the continuity of care in patients with chronic diseases [44, 47].

Physicians play a key role in providing effective continuity of care. When physicians have a continual patient–provider relationship, they are familiar with the patient’s medical history and the potential for the patient’s condition to worsen. Such physicians are more able to observe and mitigate unpredictable recurrences, and they can improve spending efficiencies by reducing unnecessary hospitalization [5, 25, 44, 48]. Promoting the roles of physicians to improve continuity of care is crucial.

The sole focus of this study was on newly diagnosed diabetic patients. Therefore, caution should be used when extending the results to other chronic diseases. Additional studies on other chronic diseases are required because of care pattern differences. Moreover, there is a risk of reverse causation. When dementia occurs, patients could be referred to new providers with expertise on such conditions. Thus, the outcome causes a reduction in continuity, rather than a lower continuity leading to a higher risk of dementia. The interpretation of study results should be made cautiously.

This study has several limitations. First, the 3-year follow-up period was short, which could probably explain the lack of an association with continuity of care regarding mortality among newly diagnosed diabetic patients. Second, this study applied claims data to conduct the analyses. However, details regarding care received and the continuity of care, including provider–patient interactions, were unavailable for evaluation. In addition, relatively little patient information was available, which could increase the risk of confounding bias. Third, this was a cross-sectional study design, which means that a correlation did not imply causation and that the risk of reverse causation could affect all of the results. Nevertheless, this study used a representative national data set to follow outpatient and inpatient care. Despite its limitations, this approach could still provide valuable insights for assessing how the continuity of care is associated with the care of newly diagnosed diabetic patients.

## Conclusions

In this study, continual care provided to newly diagnosed diabetic patients was associated with favorable health care outcomes and less medical care use. Future research might need to address the research gaps of reverse causation and omitted variable bias in order to provide stronger evidences for suggesting policy and practice change. These may help health care systems aim to enhance long-term relationships between patients and health care providers to improve the continuity of care.
